# Endovascular Salvage of Native Arteriovenous Fistulas: Post-Intervention Primary and Cumulative Patency Outcomes in a Colombian Regional Referral Center

**DOI:** 10.7759/cureus.107015

**Published:** 2026-04-14

**Authors:** Oscar F Vargas, Juliana Salcedo-Mesa, Ivan R Nieto

**Affiliations:** 1 Interventional Radiology, Hospital Departamental de Villavicencio, Villavicencio, COL; 2 Nephrology, Hospital Departamental de Villavicencio, Villavicencio, COL

**Keywords:** angioplasty, arteriovenous fistula, endovascular techniques patency, hemodialysis, ● interventional radiology, percutaneous transluminal endovascular procedure, thrombectomy, vascular

## Abstract

Introduction:* *Arteriovenous fistulas (AVFs) are the preferred vascular access for hemodialysis due to superior patency and lower complication rates. However, access dysfunction caused by stenosis and thrombosis remains frequent, requiring repeated interventions. Endovascular techniques have emerged as the first-line approach for AVF salvage, although real-world data from Latin America are limited. The objective of this study is to characterize clinical, anatomical, and procedural features of patients undergoing endovascular AVF salvage and to evaluate postintervention primary, assisted primary, and cumulative patency, as well as overall survival.

Methods: We conducted a retrospective observational study at a regional referral center in Colombia (January 2023-June 2025), including all adult patients undergoing endovascular salvage of arteriovenous fistulas (AVFs). Patients without salvage, with incomplete data, or under 18 years were excluded. Data were collected from electronic medical records and included demographic, clinical, anatomical, and procedural variables. Patency was assessed using a post-intervention approach, with time zero defined as the first endovascular procedure. Kaplan-Meier analysis was used to evaluate event-free survival (access loss or death) and overall survival, with follow-up censored at January 31, 2026. Descriptive and non-parametric statistical analyses were performed using RStudio (2024.12.1 + 563). The study was approved by the institutional ethics committee.

Results: Fifty-nine patients (mean age 59 ± 13 years; 42/59 [71.2%] male) underwent 95 interventions. Hypertension (56/59 [94.9%]) and diabetes (27/59 [45.8%]) were the most common comorbidities. Brachiocephalic fistulas (44/59 [72.9%]) and left-sided access (39/59 [66.1%]) predominated. Technical success was achieved in all patients at the index intervention (100%). The mean interval between subsequent interventions was 162 days (5.4 months). Postintervention primary patency was approximately 65-70% at 180 days and declined progressively over time. Postintervention cumulative patency remained higher, with 75% at 600 days, 65-70% at 1000 days, and 30% at 1400-1500 days. No significant differences were observed by sex or treatment technique. Overall survival remained above 80% at 600 days and decreased gradually thereafter.

Conclusions:* *Endovascular salvage of native arteriovenous fistulas is a feasible and effective strategy for preserving access function in a real-world clinical setting. Cumulative patency outcomes support the role of repeated endovascular interventions as a sustainable approach to prolonging access usability over time. While endovascular salvage appears associated with maintained patency, this relationship is likely influenced by underlying disease complexity and confounding by indication. These findings underscore the need for ongoing surveillance programs, standardized post-procedural pharmacological management, and further prospective studies to optimize long-term AVF outcomes in resource-limited settings.

## Introduction

Arteriovenous fistulas (AVFs) remain the preferred vascular access (VA) for hemodialysis due to their superior long-term patency, lower risk of infection, and improved patient survival compared to alternative modalities such as tunneled central venous catheters [1-4]. However, AVF dysfunction is a common and clinically significant problem, primarily due to stenosis and thrombosis, which may ultimately lead to access loss and increased morbidity [5,6] .

Therefore, preserving AVFs’ functionality is of clinical importance, as it is associated with improved quality of life, reduced risk of infection, and better long-term outcomes [4,7]. The management of dysfunctional AVFs has evolved over the past decades, with endovascular techniques emerging as the first-line approach for access salvage in most clinical scenarios [8,9]. 

Percutaneous interventions, including percutaneous transluminal angioplasty (PTA), thrombectomy, and stent placement, offer a minimally invasive alternative to surgical revision, with high technical success rates and the potential to preserve access function [8-10].

Despite these advances, VA dysfunction remains a chronic and recurrent condition, frequently requiring multiple reinterventions over time [4,9,11]. A major challenge in this field is the heterogeneity in reported outcomes, particularly regarding definitions of patency and access survival, which complicates comparisons across studies and limits the generalizability of findings [4]. 

To address this issue, standardized definitions have been proposed. In this study, we adopted the definitions outlined in the Society of Interventional Radiology reporting standards for hemodialysis vascular access, the “Clinical Trial End Points for Hemodialysis Vascular Access” from 2018 [12]. Postintervention primary patency was defined as the time from the index intervention to the first occurrence of access thrombosis or any reintervention aimed at maintaining or restoring patency. Postintervention assisted primary patency was defined as the interval during which the access remains functional with the aid of additional interventions and the time between each intervention. Postintervention cumulative patency was defined as the total duration of access functionality from the index intervention until access abandonment or patient death, including all intervening procedures [12].

Furthermore, most available data originate from high-income settings, with limited evidence from real-world cohorts in Latin America, where patient characteristics, healthcare access, and resource availability may differ substantially [13]. In this context, understanding the clinical behavior, procedural characteristics, and longitudinal outcomes of AVF salvage in real-world settings is essential to optimize management strategies and improve patient care [13]. 

Therefore, the aim of this study was to characterize the clinical, anatomical, and procedural features of patients undergoing endovascular salvage of arteriovenous fistulas, as well as to evaluate post-intervention primary patency, post-intervention assisted primary patency, post-intervention cumulative patency, and overall patient survival at a regional referral center in Colombia.

## Materials and methods

We conducted a retrospective, observational cohort study to characterize the clinical, anatomical, and procedural features of patients undergoing endovascular salvage of arteriovenous fistulas (AVFs), as well as to evaluate access patency and overall patient survival.

The study was performed at the Hospital Departamental de Villavicencio, a regional referral center located in the Orinoquia region of Colombia with an established interventional radiology unit, between January 1, 2023, and June 30, 2025.

All patients aged 18 years or older who underwent endovascular intervention for AVF salvage during the study period were considered eligible. Patients were excluded if endovascular salvage was not attempted due to the absence of significant stenosis, if salvage was unsuccessful and the patient was referred for surgical management or transitioned to tunneled catheter hemodialysis or peritoneal dialysis, if medical records were incomplete, or if they were under 18 years of age. Statistical analysis was performed only on AVFs that underwent successful endovascular salvage. 

A non-probabilistic consecutive sampling strategy was used, including all eligible patients within the study period. Data were collected retrospectively from electronic medical records (DINÁMICA software) and included demographic, clinical, anatomical, and procedural variables such as age, sex, comorbidities, AVF configuration and location, indication for intervention, type of endovascular technique, number of interventions, time intervals between procedures, and pharmacological treatment. Two reviewers extracted data using a standardized form with predefined variable definitions. Regarding imaging and procedural standardization of operative reports, significant stenosis was defined as luminal narrowing greater than 50%, and technical success was defined as residual stenosis less than 30% following the intervention. 

Patency was assessed using a post-intervention approach, with time zero defined as the first endovascular intervention. The interval between procedures was used to evaluate access durability, while overall access survival, including repeated interventions, was considered a measure of cumulative patency. The primary endpoints were defined as post-intervention assisted primary patency, the interval during which the access remains functional with the aid of additional interventions, and the time between each intervention, and post-intervention cumulative patency, the total duration of access functionality from the index intervention until access abandonment or patient death. Secondary endpoints were post-intervention primary patency stratified by endovascular technique and overall survival.

Statistical analysis was performed using RStudio (version 2025.09.2+418). Descriptive statistics were used to summarize the data; categorical variables were expressed as frequencies and percentages, while continuous variables were expressed as mean ± standard deviation or median with interquartile range (IQR). Normality was assessed using the Shapiro-Wilk test. Non-parametric tests, including the Kruskal-Wallis and Wilcoxon rank-sum tests, were used for group comparisons. Se usaron los siguientes paquetes estadísticos en R: "dplyr", "ggplot2", "moments", "scales", "tidyverse", "tidyr", "psych", "e1071", "survival" and "survminer." 

Kaplan-Meier survival analysis was performed to evaluate event-free survival, defined as the time from the first endovascular intervention to the occurrence of either access loss or death. Overall survival was also analyzed, with time calculated from the first intervention and patients censored at the date of death or last follow-up (January 31, 2026). The study was approved by the Ethics Committee of the Hospital Departamental de Villavicencio.

## Results

During the study period, 90 patients underwent endovascular intervention for arteriovenous fistula (AVF) salvage. Of these, 31 were excluded: 17 in whom salvage was not achieved and who were referred for surgical management or transitioned to tunneled catheter hemodialysis, eight due to missing data, one pediatric patient, one who switched to peritoneal dialysis, and four in whom no significant stenosis was identified. A total of 59 patients were included in the final analysis (Table 1).

**Table 1:** Baseline clinical characteristics Values are expressed as mean ± standard deviation (SD) or median (range), according to data distribution. Categorical variables are presented as counts and percentages. Percentages were calculated within each sex group.

Comorbidities	Male (n=42)	Female (n=17)	Total (n=59)
	n (%)	n (%)	n (%)
Hypertension	40 (95.2)	16 (94.1)	56 (94.9)
Diabetes mellitus	20 (47.6)	7 (41.2)	27 (45.8)
Coronary artery disease	9 (21.4)	4 (23.5)	13 (22)
Thrombotic risk factors	9 (21.4)	3 (17.6)	12 (20.3)
Prior thrombosis	8 (19)	2 (11.8)	10 (16.9)
Peripheral artery disease	3 (7.1)	3 (17.6)	6 (10.2)
Cardiac valvular disease	2 (4.8)	0	2 (3.4)

The mean age was 59 ± 13.2 years (range 25 to 86) with a normal distribution (Shapiro-Wilk p = 0.247). The cohort included 42 (71.2%) males and 17 (28.8%) females. Hypertension was the most common comorbidity, present in 56 (94.9%) patients, followed by diabetes mellitus in 27 (45.8%), coronary artery disease in 13 (22%), and prior thrombosis in 10 (16.9%). Valvular heart disease was uncommon, observed in 2 (3.4%) patients. Comorbidities were similarly distributed between sexes. However, valvular disease was only observed in males, while peripheral arterial disease was more frequent in females (3 patients, 17.6%) (Table 1).

In terms of AVF characteristics, the most common configuration was brachiocephalic in 44 (72.9%) cases, followed by radiocephalic in 8 (13.6%). Left-sided access was more frequent, accounting for 66.1% of cases (Table 2).

**Table 2:** Anatomical configuration of arteriovenous fistulas Values are expressed as counts and percentages. Percentages for each anatomical configuration are calculated relative to the total number of patients in the study cohort. Laterality (right and left) is reported as a proportion of the overall sample. (n=59)

Anatomical Configuration	Total	Right n (%)	Left n (%)
Brachiocephalic	44 (72.9)	14 (23.7)	30 (50.9)
Radiocephalic	8 (13.6)	5 (8.5)	3 (5.1)
Brachiobasilic	4 (6.8)	0	4 (6.7)
Brachiobrachial	3 (5.1)	1 (1.7)	2 (3.4)

**Figure 1:**
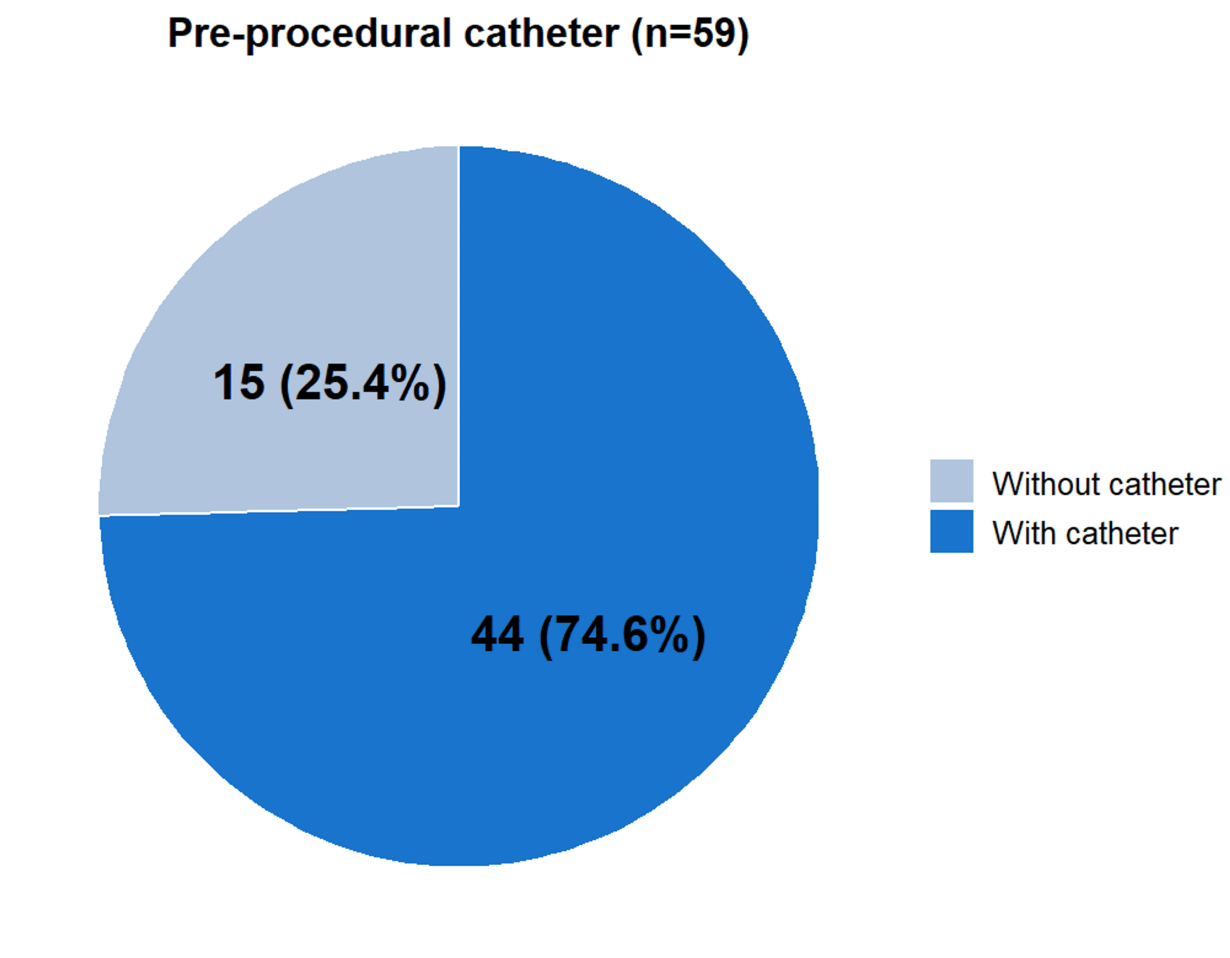
Pre-procedural catheter status among patients undergoing AVF salvage Distribution of patients according to the presence or absence of a catheter prior to the first endovascular intervention. Most patients (74.6%) had a catheter in place before the procedure, whereas 25.4% underwent intervention without a pre-existing catheter.

A total of 59 patients underwent 95 interventions. Most patients required a single intervention (33 patients, 55.9%), while 18 (30.5%) underwent two procedures. Fewer patients required three (6 patients, 10.2%) or four (2 patients, 3.4%) interventions (Table 3). The median time to the first intervention, defined as the interval between initial access dysfunction and the first procedure, was seven days (IQR: 4.5 to 12.5). (Table 4) The most common indication for the first intervention was thrombosis (62.7%, n=37), followed by stenosis (31.5%, n=18). Angioplasty alone was the most frequently performed procedure (35.6%, n=21). Prior to endovascular salvage, 44 patients (74.6%) had a tunneled hemodialysis catheter (Figure 1). All AVFs were successfully salvaged after the first intervention in the included cohort (Table 4).

**Table 3:** Number of endovascular procedures per patient Values are expressed as counts and percentages of patients undergoing one or more endovascular procedures. Percentages were calculated relative to the total number of patients included in the study (n=59).

Procedures Per patient	n (%)
1	33 (55.9)
2	18 (30.5)
3	6 (10.2)
4	2 (3.4)

**Table 4:** Procedural characteristics, indications, and outcomes across sequential endovascular interventions Continuous variables are expressed as medians (interquartile range [IQR]), and categorical variables as counts and percentages. Percentages are calculated relative to the total number of procedures at each intervention stage. Time intervals between procedures are reported in days, with the first interval corresponding to the time from the index intervention to the first reintervention. AVF: arteriovenous fistula. * The time between first access dysfunction and first endovascular salvage.

Variable	Intervention 1 (n=59)	Intervention 2 (n=26)	Intervention 3 (n=8)	Intervention 4 (n=2)
Time between Interventions, days, Median (IQR)	Median 7 days (IQR: 4.5–12.5)*	Median 171.5 days (IQR: 84.3–384.8)	Median 153 days (IQR: 3–193.5)	Median 163 days (IQR: 153.5–172.5)
Indication for Intervention (AVF dysfunction)				
Stenosis	11 (18.6)	3 (11.5)	1 (12.5)	-
Thrombosis	37 (62.7)	19 (73.1)	7 (87.7)	2 (100)
Stenosis + Thrombosis	9 (15.3)	2 (7.7)	-	-
Stenosis + Pseudoaneurysm	1 (1.7)	1 (3.9)	-	-
Steal syndrome	1 (1.7)	-	-	-
Infection	-	1 (3.9)	-	-
Type of procedure performed				
Angioplasty	21 (35.6)	15 (57.7)	3 (37.5)	2 (100)
Angioplasty + Stent	5 (8.5)	2 (7.7)	1 (12.5)	-
Thrombectomy	1 (1.7)	1 (3.9)	2 (25)	-
Thrombectomy + Angioplasty	22 (37.3)	3 (11.5)	-	-
Thrombectomy + Angioplasty + Stent	10 (16.9)	1 (3.9)	-	-
Procedure not feasible	-	4 (15.4)	2 (25)	-
AVF Outcome				
Fistula salvaged	59 (100)	22 (84.6)	6(75)	2(100)
Fistula loss	-	4 (15.4)	2(25)	-

**Figure 2:**
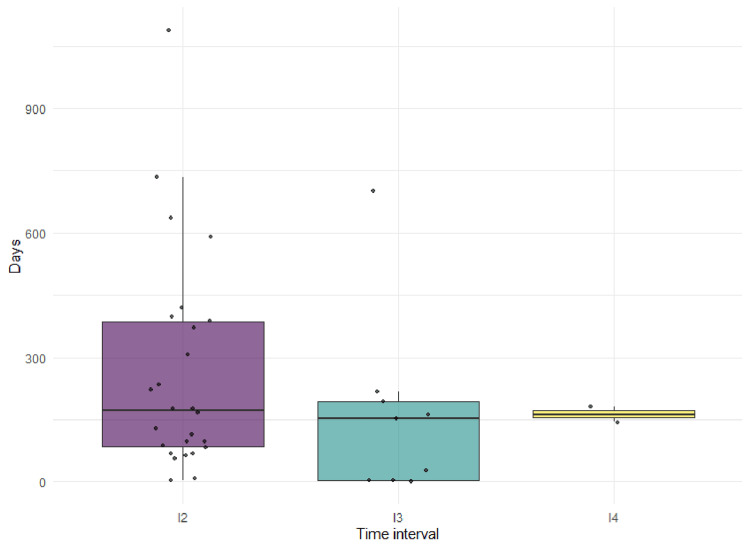
Time interval between sequential endovascular interventions (n=59) Boxplot illustrating the time intervals (in days) between sequential endovascular interventions for arteriovenous fistula (AVF) dysfunction. A shorter interval to the first reintervention (I2) compared with subsequent procedures (I3 and I4) suggests early recurrence of access dysfunction following the index procedure. The median is shown by the central line, with boxes representing the interquartile range and individual observations displayed as points.

A total of 26-second interventions were performed. The median time between the first intervention and reintervention was 171.5 days (IQR 84.3-384.8) and 153 days (IQR 3-193.5) between the second and third procedures (Figure 2). The mean time between interventions two, three, and four was 162 days (5.4 months). The interval between procedures was used as a surrogate indicator of assisted primary patency rather than a formal estimate based on standardized event-time definitions. Thrombosis was the most frequent indication for reintervention. Angioplasty, either alone or in combination with thrombectomy, remained the most commonly performed technique. Although most fistulas were successfully salvaged, the proportion of fistula loss increased with subsequent interventions (Table 4). Additionally, the most frequently affected anatomical site was the cephalic vein, observed in 16 of 59 cases (27.1%), followed by lesions in the innominate vein in 9 of 59 cases (15.3%), and the basilic vein in 3 of 59 cases (5.1%). The remaining data could not be captured due to incomplete anatomical documentation.

Pharmacological treatment following the initial intervention was heterogeneous, given the absence of standardized institutional guidelines. Treatment selection was based on the treating specialist's clinical judgment. Anticoagulation monotherapy and dual antiplatelet therapy were the most frequently prescribed regimens, each administered to 17 patients (28.8%) (Table 5).

**Table 5:** Pharmacological treatment regimens following endovascular intervention Values are expressed as counts and percentages of patients receiving each pharmacological regimen following endovascular intervention. Percentages were calculated based on the total study population. ASA: acetylsalicylic acid.

Pharmacological Treatment Regimens	n (%)
ASA alone	7 (11.9)
Clopidogrel alone	1 (1.69)
Anticoagulation alone	17 (28.8)
ASA + Anticoagulation	6 (10.2)
Dual antiplatelet therapy	17 (28.8)
Dual antiplatelet therapy + Anticoagulation	8(13.6)
No pharmacological treatment	3 (5.1)

Arteriovenous fistula patency

Postintervention Primary Patency

Kaplan-Meier analysis demonstrated a progressive decline in postintervention primary patency over time. At baseline, all accesses were patent (100%). A marked reduction in patency was observed during the early follow-up period, with a steep decline within the first 100-200 days.

Estimated primary patency decreased to approximately 70-75% in around 100 days and to nearly 60% at 400-500 days. This decline continued gradually over time, reaching approximately 50% at around 900-1000 days. At longer follow-up, primary patency further decreased to approximately 35-40% beyond 1200-1400 days.

**Figure 3:**
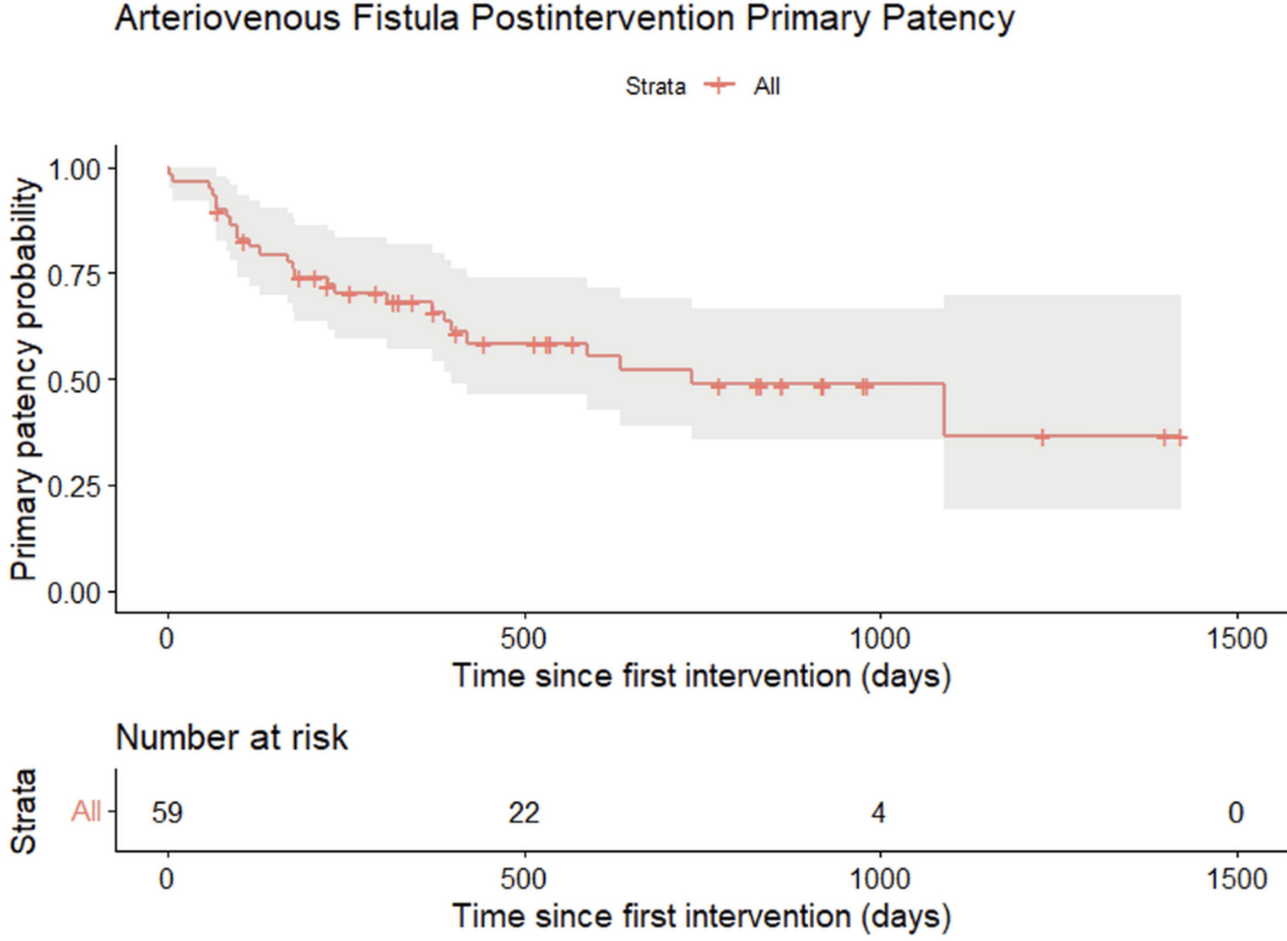
Postintervention primary patency Kaplan–Meier estimate of primary patency after endovascular intervention for AVF dysfunction. Results are shown for the overall cohort (strata: All), with shaded areas representing 95% confidence intervals.

The number of patients at risk decreased over time, from 59 at baseline to 22 at 500 days and 4 at 1000 days, with no patients remaining at risk beyond 1500 days. Confidence intervals widened at later time points, reflecting increased uncertainty due to the reduced number of patients under observation (Figure 3).

Postintervention Cumulative Patency

**Figure 4:**
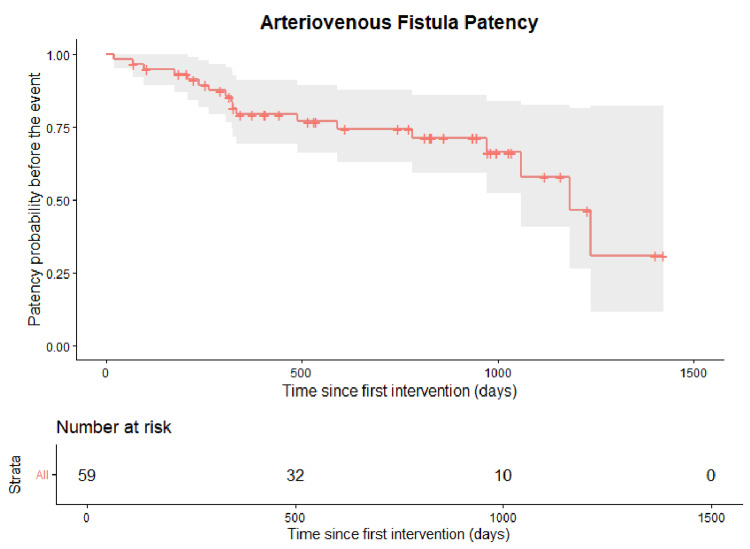
Postintervention cumulative patency (n=59) Kaplan–Meier estimate of postintervention cumulative AVF patency after endovascular intervention (strata: all). The event was defined as vascular access loss or patient death. Shaded areas indicate 95% confidence intervals.

Kaplan-Meier analysis demonstrated a progressive decline in event-free survival over time. At baseline, all patients were free of the endpoint (death or access loss). The probability of remaining event-free decreased gradually, with an estimated patency of approximately 75% at around 600 days, 65-70% at 1000 days, and close to 30% at 1400-1500 days of follow-up (Figure 4).

**Figure 5:**
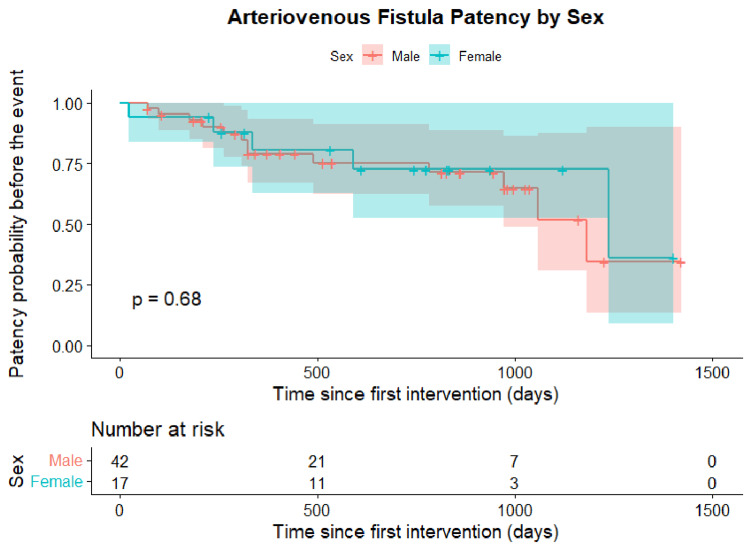
Postintervention cumulative patency stratified by sex (n=59) Kaplan–Meier curves showing cumulative patency of arteriovenous fistulas (AVFs) stratified by sex. The event was defined as vascular access loss or patient death. Differences between groups were assessed using the log-rank test (p=0.68). Shaded areas represent 95% confidence intervals.

Furthermore, Kaplan-Meier analysis stratified by sex showed no significant differences in event-free survival between male and female patients (log-rank p = 0.68). Both groups demonstrated a similar progressive decline in patency over time, with overlapping survival curves throughout the follow-up period (Figure 5).

**Figure 6:**
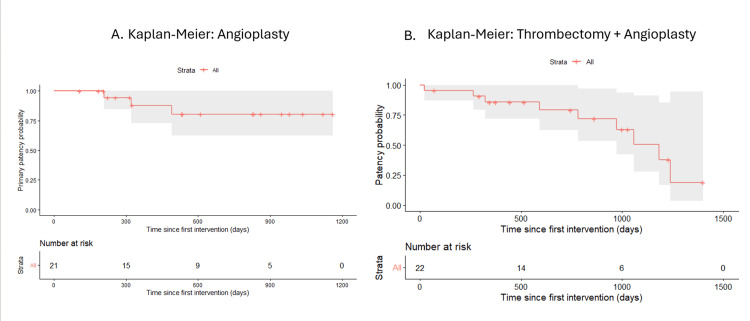
Kaplan-Meier estimates of cumulative patency of arteriovenous fistulas (AVFs) stratified by endovascular treatment modality (A) Angioplasty alone (n=21). Cumulative patency remained above 80% throughout the observation period, with 15 patients at risk at 300 days, 9 at 600 days, and 5 at 900 days. The curve demonstrates sustained access functionality with minimal decline beyond the initial follow-up period. (B) Thrombectomy combined with angioplasty (n=22). Cumulative patency showed progressive decline, starting above 90% and decreasing to approximately 75% at 500 days, 50% at 1000 days, and below 25% at 1500 days. Patients at risk: 14 at 500 days and 6 at 1000 days. The event was defined as vascular access loss or patient death. Shaded areas indicate 95% confidence intervals. Censored observations are indicated by vertical tick marks. Risk tables are displayed below each curve.

**Figure 7:**
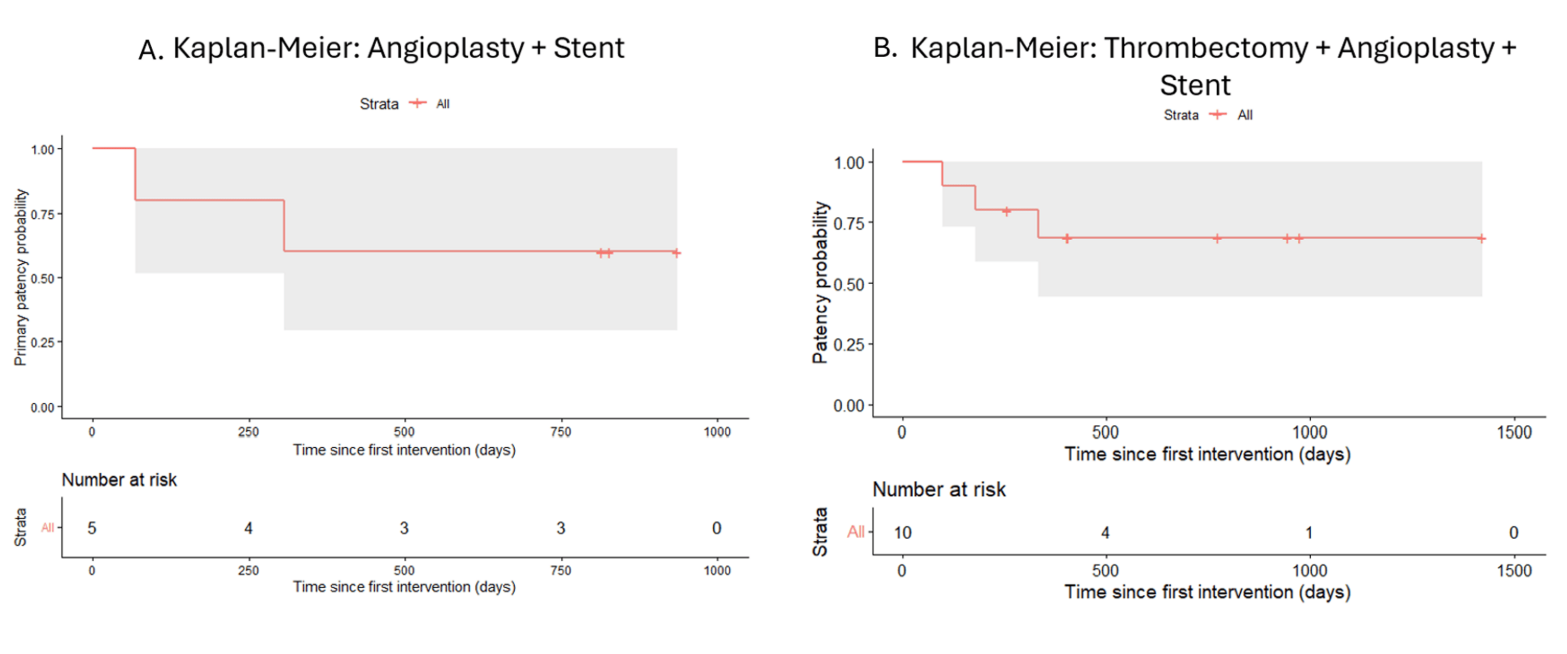
Kaplan-Meier estimates of cumulative patency of arteriovenous fistulas (AVFs) stratified by endovascular treatment modality (A) Angioplasty plus stent (n=5). Cumulative patency decreased from 100% to approximately 80% within the first 250 days, then stabilized at approximately 60% from 300 days through 1000 days. Patients at risk: five at baseline, four at 250 days, three at 500 days, and three at 750 days. (B) Thrombectomy combined with angioplasty plus stent (n=10). Cumulative patency showed a stepwise decline, decreasing from 100% to approximately 80% at 200 days, then to approximately 65% at 500 days, remaining stable through 1500 days. Patients at risk: 10 at baseline, four at 500 days, and one at 1000 days. The event was defined as vascular access loss or patient death. Shaded areas indicate 95% confidence intervals. Censored observations are indicated by vertical tick marks. Risk tables are displayed below each curve.

Moreover, when analyzed by treatment techniques, it showed differences in event-free survival across groups. Patients treated with angioplasty alone (n=21) demonstrated the most favorable patency profile, with a relatively stable curve over time. In contrast, those undergoing thrombectomy combined with angioplasty (n=22) exhibited a more pronounced decline in patency, particularly at longer follow-up. Patients treated with combined approaches, including stent placement, showed intermediate outcomes, including those undergoing thrombectomy, angioplasty, and stent placement (n=10). The angioplasty plus stent group (n=5) also showed a decline in patency; however, interpretation in this subgroup is limited due to the small sample size (Figures 6, 7).

Overall survival

**Figure 8:**
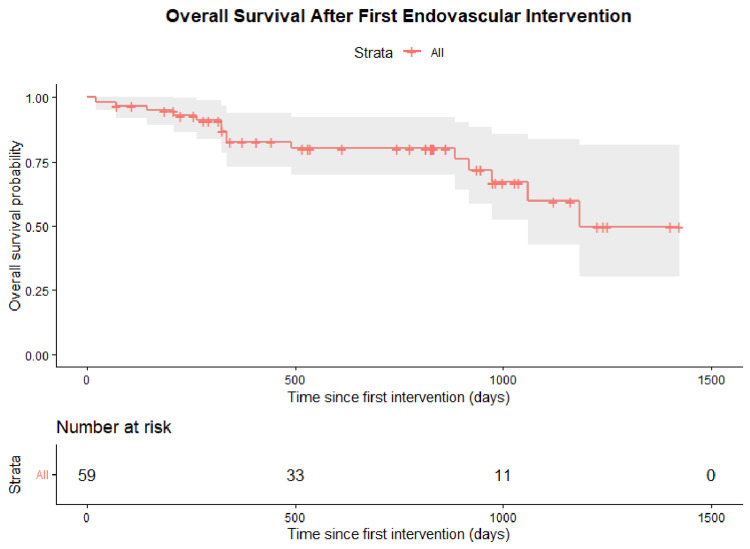
Overall patient survival (n=59) Kaplan–Meier estimate of overall survival after first endovascular intervention (strata: All). Shaded areas indicate 95% confidence intervals.

Overall survival also showed a gradual decline following the first intervention. Survival remained above 80% at approximately 600 days, decreased to 65-70% at 1000 days, and reached approximately 50% at 1400-1500 days of follow-up (Figure 8).

Finally, non-parametric analyses showed no significant differences in total patency according to treatment technique (Kruskal-Wallis p = 0.218) or in the number of reinterventions across procedural groups (p = 0.203). In exploratory analyses, patients with peripheral arterial disease exhibited a longer median patency compared to those without (972 vs. 307 days), although this difference did not reach statistical significance (Mann-Whitney U test, p = 0.076) (Tables 6, 7).

**Table 6:** Association between procedural technique and clinical outcomes (N=59) Values expressed as median (interquartile range). PTA: percutaneous transluminal angioplasty. The Kruskal-Wallis H test is a non-parametric method used to compare medians across three or more independent groups when data do not follow a normal distribution; a p-value <0.05 indicates statistically significant differences between groups.

Procedural Technique	n	Total Patency (days), median (IQR)	Reinterventions, median (IQR)	Kruskal–Wallis H (Patency)	p-value (Patency)	Kruskal–Wallis H (Reinterventions)	p-value (Reinterventions)
PTA alone	21	322 (267–406)	1 (1–1)	5.75	0.219	5.95	0.203
PTA + Stent	5	188 (128–248)	2 (1–2)				
Thrombectomy alone	1	237 (NA)	3 (NA)				
Thrombectomy + PTA	22	782 (323–1058)	1 (1–2)				
Thrombectomy + PTA + Stent	10	177 (138–256)	1 (1–1.75)				

**Table 7:** Association between peripheral arterial disease and total patency (N=59) Values expressed as median (interquartile range). The Mann-Whitney U test is a non-parametric method used to compare two independent groups when data do not follow a normal distribution; a p-value <0.05 indicates a statistically significant difference between groups. PAD: peripheral arterial disease.

PAD Status	n (%)	Total Patency, days	Mann-Whitney U	p-value
No	53 (89.8)	307 (193–541)	7	0.076
Yes	6 (10.2)	972 (654–1105)		

## Discussion

This study provides a comprehensive characterization of patients undergoing endovascular salvage of AVFs in a real-world setting. Our findings support the effectiveness of endovascular techniques as a first-line strategy for access preservation, while acknowledging the chronic and progressive nature of vascular access dysfunction.

Regarding baseline characteristics, our cohort was comparable to previously reported populations, with a mean age of 56 ± 13 years, similar to other studies reporting a mean age of 57 (±13) years [14,15]. Hypertension was the most common comorbidity, followed by diabetes mellitus and coronary artery disease, consistent with prior reports [16].

These findings are clinically relevant, as factors such as advanced age, male sex, obesity, and a Charlson comorbidity index >7 have been associated with reduced patency at one year [17,18]. However, in our cohort, no statistically significant associations were identified.

Regarding anatomical configuration, our results were also consistent with the literature, with left-sided and brachiocephalic fistulas being the most common, followed by radiocephalic access, as previously reported [16].

In our cohort, endovascular salvage was successful in all patients during the initial intervention, supporting prior evidence that minimally invasive approaches represent a safe and effective alternative to surgical management [9]. These results are consistent with previous studies reporting high technical and clinical success rates for endovascular treatment of dysfunctional AVFs. In our cohort, estimated postintervention primary patency was approximately 65-70% at 180 days (6 months), decreasing to nearly 60% at 400-500 days (13-17 months), with a continued gradual decline over time. Postintervention primary patency reached approximately 50% at around 900-1000 days (30-33 months) and further decreased at longer follow-up. These findings are better than other studies where post-intervention primary patency at 360 days was 30.2% [19]. However, our results are consistent with a prior study, where post-thrombectomy primary and assisted primary were 62.9% at three months and 59.5% at six months, respectively [20]. This pattern reflects the recurrent stenosis and thrombosis that necessitate repeated interventions.

The analysis of time between interventions was used in this study as a surrogate marker of postintervention assisted primary patency. The mean time between each intervention, two, three, and four, was 162 days (5.4 months). The analysis of time between interventions, used in this study as a surrogate marker of postintervention-assisted primary patency, further supports this concept. The mean time between the second, third, and fourth interventions was 162 days (5.4 months). This is important, as it suggests that some patients will require periodic maintenance of the vascular access to preserve its functionality over time. Additionally, knowing this interval is useful to estimate and plan follow-up and surveillance strategies, allowing timely intervention before irreversible access damage occurs and salvage is no longer feasible.

Importantly, postintervention cumulative patency outcomes were more favorable, with approximately 75% of patients remaining free of access loss or death at around 600 days (20 months), decreasing to 65-70% at approximately 1000 days (33 months), and to nearly 30% at 1400-1500 days (46-50 months) of follow-up. As mentioned previously, finding comparable metrics across studies remains challenging, particularly when contrasted with secondary patency, which is defined from the time of fistula creation until access loss or death. In one study, secondary patency at one year (12 months) was reported as 59.3%, which appears lower compared to our findings [19]. In another study, secondary patency at three years was reported as 66.5% (95% CI, 63.6%-69.5%), which may be comparable to our findings [15]. However, an Indian cohort reported higher secondary patency rates of up to 94% at 24 months [21]. These comparisons should be interpreted with caution due to the variability in patency definitions across studies. In our study, true secondary patency could not be assessed, as the date of fistula creation was not available; instead, we report postintervention cumulative patency. These findings reinforce the clinical value of repeated endovascular interventions in prolonging access usability over time.

When outcomes were analyzed according to treatment technique, angioplasty alone appeared to be associated with better patency compared to more complex interventions such as thrombectomy or stent placement [6]. However, these findings should be interpreted with caution, as the choice of technique is inherently influenced by the number of these procedures and lesion severity [4,22]. Patients requiring thrombectomy or stent placement likely represent a subgroup with more advanced or complex disease, which may explain the poorer outcomes observed [6,23,24]. Therefore, the differences in patency across techniques may reflect underlying disease severity rather than the intrinsic effectiveness of each approach.

An important challenge in AVF management is the lack of consensus regarding pharmacological therapy following endovascular intervention, particularly according to the underlying etiology. Evidence remains limited and is largely derived from studies on grafts. In this setting, dual antiplatelet therapy has been associated with reduced loss of primary patency, although combined aspirin and anticoagulation may be associated with worse survival [25]. A recent meta-analysis also suggests that antithrombotic and antiplatelet therapies may improve patency outcomes [26]. In our cohort, post-procedural pharmacological strategies were heterogeneous, reflecting this lack of standardization and patient complexity.

Maintaining a functional arteriovenous fistula is essential, as it improves quality of life and supports treatment continuity in patients on hemodialysis. In our cohort, overall survival remained relatively stable early on and declined gradually over time, likely reflecting the burden of comorbidities rather than the intervention itself. Chronic kidney disease is a major contributor to global mortality, particularly cardiovascular death, and outcomes in dialysis patients are influenced not only by non-modifiable factors but also by vascular access [27,28]. Although endovascular interventions are key to maintaining access function, their direct impact on survival cannot be established in this study.

Finally, although no statistically significant differences were observed in patency according to treatment technique, exploratory findings suggested longer patency in patients with peripheral arterial disease. This unexpected finding observation did not reach statistical significance and should be interpreted cautiously, but it may warrant further investigation in larger studies.

This study has several limitations. Its retrospective, single-center design may introduce selection and ascertainment bias, compounded by incomplete anatomical documentation in some cases. No formal power calculation was performed, and the relatively small sample size limits statistical power, particularly for subgroup analyses; therefore, these findings should be considered exploratory and underpowered. The lack of data on the date of fistula creation precludes assessment of true secondary patency, and defining time zero at the index intervention may introduce immortal time bias. Confounding by indication is an important consideration, as the choice of endovascular technique was inherently driven by lesion severity, and the heterogeneity of post-procedural pharmacological regimens represents an additional unmeasured confounder. The absence of multivariable adjustment limits the ability to identify independent predictors of patency or survival. Furthermore, surveillance bias may have influenced reintervention rates, and loss to follow-up was not formally distinguished from administrative censoring, which may affect the reliability of later Kaplan-Meier estimates. As a single-center study from the Orinoquia region of Colombia, generalizability may be limited given differences in patient demographics, healthcare access, and referral pathways.

Nevertheless, this study provides valuable real-world data and contributes to the growing body of evidence supporting endovascular techniques in AVF management. Prospective studies with adequate sample sizes and multivariable adjustment are warranted to better characterize patterns of access dysfunction, evaluate outcomes by endovascular technique, and determine optimal pharmacological management following AVF salvage. 

## Conclusions

Endovascular salvage of native arteriovenous fistulas maintains access function in real-world settings, with preserved cumulative patency supporting the feasibility of repeated interventions. While these findings suggest that ongoing endovascular management may contribute to sustained access function, the observational nature of this study and potential confounding by disease complexity preclude definitive causal inferences. These results highlight the need for ongoing surveillance and further prospective studies to optimize management strategies.
